# Bacterial Spore Inactivation in Orange Juice and Orange Peel by Ultraviolet-C Light

**DOI:** 10.3390/foods10040855

**Published:** 2021-04-15

**Authors:** Pilar Colás-Medà, Iolanda Nicolau-Lapeña, Inmaculada Viñas, Isma Neggazi, Isabel Alegre

**Affiliations:** AGROTECNIO-CERCA Center, Food Technology Department, Universitat de Lleida, Rovira Roure 191, 25198 Lleida, Spain; pilar.colas@udl.cat (P.C.-M.); iolanda.nicolau@udl.cat (I.N.-L.); inmaculada.vinas@udl.cat (I.V.); isma.neggazi@udl.cat (I.N.)

**Keywords:** *Alicyclobacillus acidoterrestris*, *Bacillus cereus*, *Bacillus coagulans*, UV-C radiation, UV-C light, inactivation, surface, fruit, predictive model, Weibull

## Abstract

Spore-forming bacteria are a great concern for fruit juice processors as they can resist the thermal pasteurization and the high hydrostatic pressure treatments that fruit juices receive during their processing, thus reducing their microbiological quality and safety. In this context, our objective was to evaluate the efficacy of Ultraviolet-C (UV-C) light at 254 nm on reducing bacterial spores of *Alicyclobacillus acidoterrestris*, *Bacillus coagulans* and *Bacillus cereus* at two stages of orange juice production. To simulate fruit disinfection before processing, the orange peel was artificially inoculated with each of the bacterial spores and submitted to UV-C light (97.8–100.1 W/m^2^) with treatment times between 3 s and 10 min. The obtained product, the orange juice, was also tested by exposing the artificially inoculated juice to UV-C light (100.9–107.9 W/m^2^) between 5 and 60 min. A three-minute treatment (18.0 kJ/m^2^) reduced spore numbers on orange peel around 2 log units, while more than 45 min (278.8 kJ/m^2^) were needed to achieve the same reduction in orange juice for all evaluated bacterial spores. As raw fruits are the main source of bacterial spores in fruit juices, reducing bacterial spores on fruit peels could help fruit juice processors to enhance the microbiological quality and safety of fruit juices.

## 1. Introduction

The microbial population of fruit and vegetable juices depends on the agricultural practices used to obtain the raw materials, the employees’ health and hygiene and the source of agricultural water, as well as on the production practices and the hygienic conditions of the processing plant. The utilization of fruit that has fallen to the ground can introduce additional microorganisms due to their contact with the soil. Traditionally, foodborne disease outbreaks have not been associated with fruit juices mainly because of their low pH values (pH < 4.5) [1] and the pasteurization or the high hydrostatic pressure (HPP) treatment that they receive. However, nowadays, a non-depreciable number of outbreaks have been linked to raw juice consumption, being *Escherichia coli* O157:H7 and *Salmonella* the main pathogens implicated [2]. On the contrary, spoilage microorganisms have always been a matter of great concern for the stability of fruit juices as the heat treatment they usually receive is not enough to control heat-resistant ascospores from fungi and bacterial spore-formers [3]. 

Although thermal pasteurization is widely used during fruit and vegetable juice processing to ensure its microbial safety and quality, thermal treatments are detrimental for juice organoleptic and nutritional quality. Therefore, some non-thermal pasteurization methods have been proposed during the last decades, including HPP, pulsed electric fields, ultrasound and ultraviolet light [4]. Currently, HPP is widely used to pasteurize juices of superior quality as it does not produce heat-related deterioration like loss of vitamins and nutrients, discoloration and textural and organoleptic changes [5]. Both thermal pasteurization and HPP treatments of fruit and vegetable juices are able to inactivate bacterial vegetative forms. Despite this, bacterial spores are more resistant. For example, they have been reported to withstand at least 1000 MPa, which is 400 MPa higher than the maximum pressure currently achievable in commercial food processing [6].

*Alicyclobacillus* is a thermoacidophilic spore-forming bacterium that can spoil acidic juices as it is able to grow at a temperature range from 20–70 °C and at a pH from 2.5–6.0 [7]. Since it was first identified as a spoilage bacterium for fruit juices [8], it has become a major concern for the juice industry. In the survey conducted by Snyder and Worobo [9], 78% of juice processors reported being concerned about *Alicyclobacillus* contamination. Fruit juice spoilage caused by *Alicyclobacillus* species is difficult to detect by fruit juice processors as it does not produce gas or any other visible changes in the product. Sometimes, consumers’ complaints are the only way for companies to become aware of the problem. *Alicyclobacillus* causes off-flavours in the odour and taste of the spoiled product that has been described as medicinal, disinfectant-like or antiseptic due to the production of guaiacol and other chemical compounds, such as the halophenols 2,6-dichlorophenol and 2,6-dibromophenol [10]. *Alicyclobacillus acidoterrestris* is the main species implicated in juice spoilage [11]. Soil is considered to be the main habitat of *A. acidoterrestris* and also the most important source of contamination of acidic products [12]. Studies have suggested that the contamination of fruit juices is most likely caused by fruit contamination by soil during harvest or by unwashed or poorly washed raw fruit used in processing facilities [7]. 

Other spore-forming bacteria genera have been isolated from fresh fruits and their juices, such as *Bacillus coagulans* and *Bacillus cereus*. *B. coagulans* causes flat sour-type spoilage in heat-processed acid beverages, tomato juice and acidified vegetable products because they form heat-resistant spores that can grow at low pH. This organism is frequently isolated from spoiled canned vegetables acidified to pH 4.0–4.5 and has been considered the primary cause of economically important spoilage in thermally processed tomatoes and tomato products [13,14]. Although *B. coagulans* is a non-pathogenic microorganism, it might cause a food safety hazard due to its ability to increase the pH of acidic foods to a level that can allow the germination of surviving *Clostridium botulinum* spores [13,15].

Though *B. cereus* is a ubiquitous bacterium, the soil is the main source of its spores. Therefore, its presence in most raw foods is inevitable. Moreover, the spores have strong adhesion properties, so additional contamination may occur due to biofilms formed on the surface of processing equipment. This bacterium has been isolated in almost every category of foodstuffs [16], including juices. For example, in the study carried out by Aneja et al. [17], *B. cereus* was detected in 56.7% of the studied unpasteurized juices. In another study, *B. cereus* was observed in 64.9% of the samples of unpasteurized street-vended fruit juices [18]. Although *B. cereus* is not particularly tolerant to acid, orange juice has been implicated in *B. cereus* foodborne poisoning [19]. 

As current techniques used for juice production do not control spore-forming bacteria, other strategies and treatments are under study. For example, Roig-Sagués et al. [20] combined preheating the samples at around 80 °C with an ultra-high pressure homogenization at 300 MPa to achieve a 5 log reduction of the *A. acidoterrestris* population. Short-wave ultraviolet-C (UV-C) light radiation in the range of 200–280 nm, which mainly breaks down DNA molecules resulting in a germicidal effect on bacteria, virus, protozoa, fungi and algae [21], has been approved by the FDA to treat food surfaces and fruit juices [22]. However, the main limiting factor in UV-C technology is the low-penetration capacity in liquid foods with a high absorption coefficient [23]. Juice treatment with UV-C radiation does not produce toxic by-products, and it does not change their taste or colour profiles. Differently to other non-thermal technologies, UV-C is capable of inactivating bacterial spores [24], and it has been previously described to reduce *A. acidoterrestris* spores in white grape, apple, ‘Cantaloupe’ melon and orange juices with treatments ranging from 8 to 20 min at UV-C intensities among 1.31–14 W/m^2^ [21,25,26,27]. However, Baysal et al. [25] found that the reduction of *A. acidoterrestris* population of spores depended on the UV dose and fruit juice type, observing a higher reduction of bacterial spores in white grape juice (5.5 log CFU/mL reduction) when compared to apple juice (2.1 log CFU/mL) at the same UV-C intensity (1.31 mW/cm^2^). The difference in UV-C treatment effectiveness was given by the lower absorption coefficient of the tested grape juice (5.82 cm^−1^) when compared to apple juice (12 cm^−1^). The reduction of *B. cereus* and *B. coagulans* spores by UV-C treatment has been reported in synthetic media [28], but no references have been found in juices. 

As the efficacy of UV-C treatment on fruit juices depends on the transmittance of the juice and considering that *Alicyclobacillus* spp. and other spore-forming bacteria are soil-borne, their control should start with proper cleaning of the fruits at the beginning of processing [29]. Therefore, the aim of this study is to determine the efficacy of UV-C radiation at 254 nm to reduce the spores of *A. acidoterrestris*, *B. coagulans* and *B. cereus* at two stages of orange juice production. Specifically, the application of this technique on the whole fruit (orange peel) and after obtaining the juice (orange juice) is studied. 

## 2. Materials and Methods

### 2.1. Bacterial Spore Production

The strains used in this investigation, *Alicyclobacillus acidoterrestris* (CECT 7094T, isolated from a garden soil), *Bacillus cereus* (CECT 131, isolated from a contaminated flask) and *Bacillus coagulans* (CECT 561, unknown origin), were provided by the Spanish Type Culture Collection (CECT). In addition, another *A. acidoterrestris* strain, TA-1.183, isolated from a pasteurized apple juice sample in our laboratory, was used. This strain was identified by 16S rDNA gene sequencing and included in the Food Technology Department of the University of Lleida collection. All the strains were stored in 30% glycerol at −20 °C. 

The *A. acidoterrestris* strains were streaked onto Yeast Starch Glucose Agar ((YSG), Scharlau, Spain) plates and incubated at 45 °C for 24 h. The *Bacillus* spp. strains were streaked onto nutrient agar ((NA), Biokar, France) plates and incubated at 30 °C for 24 h. To obtain the vegetative cultures of the *A. acidoterrestris* strains, a single colony was inoculated into 10 mL of *Bacillus acidoterrestris* broth ((BAT broth), Scharlau) and incubated at 45 °C for 24 h. For the *Bacillus* spp., a single colony was inoculated into 10 mL of Tryptic Soy Broth ((TSB), Biokar) and incubated at 30 °C for 24 h. 

To induce sporulation of the *A. acidoterrestris* strains, the cells grown at 45 °C for 24 h on BAT broth were spread onto YSG agar and incubated at 45 °C for seven days until at least 80% of the cells sporulated, according to Baysal et al. [25]. For the *Bacillus* spp., the cells grown at 30 °C for 24 h on TSB were spread onto NA supplemented with MnSO_4_·7H_2_O (50 mg/mL) (NAMS) and incubated at 30 °C for five days until at least 80% of the cells sporulated, according to Beuchat et al. [30]. All bacterial spores were harvested as described by Baysal et al. [25]. Briefly, a total of 5 mL of sterile deionized water was placed onto the surfaces of YSG and NAMS agar and then dislodged by gentle rubbing with a sterile swab. Suspensions of each strain were centrifuged (3000× *g*, 15 min 20 °C, Hettich-Universal 320 R, Tuttlingen, Germany) and resuspended in sterile deionized water. The washing procedure was repeated four times. The final pellets were resuspended in sterile deionized water and stored at 4 °C until use. The viability of *A. acidoterrestris* spore suspensions was determined by dilution in saline peptone solution ((SP), 1.0 g/L peptone (Biokar) and 8.5 g/L NaCl (VWR chemicals, Radnor, PA, USA)), activated by heat shock (80 °C for 12 min in a water bath), serially diluted in SP, spread onto a YSG agar plates and incubated at 45 °C for 24 h. The viability of *B. cereus* and *B. coagulans* spore suspensions was determined by dilution in SP, activated by heat shock (80 °C for 10 min in a water bath), serially diluted in SP, spread onto an NA plates and incubated at 30 °C for 24 h.

### 2.2. Orange and Juice Samples

The oranges were purchased from a local supermarket, and the pasteurized orange juice was provided by a juice industry located in Reus (Spain). The absence of *A. acidoterrestris* in the orange juice was confirmed according to IFU Method No. 12:2019; meanwhile, the absence of *Bacillus* spp. spores was confirmed by plating a sample of juice after a heat shock. The pH values of the juice were measured by a pH meter (XS Instruments, Carpi, Italy). The soluble-solid content (SSC) was determined using a handheld refractometer (PAL-1, Atago, Japan), and the results were expressed in °Brix. To measure the titratable acidity (TA), 10 mL of orange juice was diluted with 10 mL of deionized water and titrated with a sodium hydroxide (NaOH, 0.1 N) solution to pH 8.1. The results were calculated as g of citric acid per L of juice. The UV light transmittance at 254 nm was measured using a spectrophotometer (UV-1600PC, VWR International, Radnor, PA, USA). The sample’s transmittance as percent transmittance, which is related to absorbance (A) by Equation (1), was evaluated using a quartz cuvette with path lengths of 1.0 cm.

(1) $$A=-{log}_{10}\;\;\left(\frac{\%T}{100}\right)$$

### 2.3. Sample Inoculation

Prior to orange peel inoculation, the surface wax was washed off by immersing the fruit in hot water (70 °C) for 30 s and dried at room temperature, according to Yoo et al. [31]. After that, peel orange plugs (2.54 cm^2^/plug) were cut using a cork borer. Each peel plug was spot inoculated with 0.05 mL of spore suspension (6 × 10^6^ CFU/mL) to obtain a population of 5.0 log CFU/cm^2^. Peel plugs were kept at room temperature for 2 h before UV-C treatment. Orange juice was inoculated with the spore suspension to obtain an initial concentration of 5.0 log CFU/mL. The bacterial spore population was checked as described in Section 2.5.

### 2.4. UV-C Device and Inactivation Treatments

UV-C treatments were carried out in laboratory scale equipment manufactured by LAB-UVC-Gama (Steribelt, UV-Consulting Peschl España, Geldo, Spain). The camera (61.8 × 27.7 × 20 cm) was equipped with three monochromatic UV-C lamps emitting at 254 nm (UV 254 nm, 30 W/lamp). The samples were positioned at a 12 cm distance from the lamps. The intensity of UV-C during the treatments was monitored using a radiometer UV-sensor Easy H1 (Peschl Ultraviolet, Mainz, Germany) placed at the same distance from the lamps as the samples. Before the experiment started, UV-C lamps were switched on for 30 min to ensure light stability. 

Orange inoculated peel plugs were placed in a petri dish and treated by UV 254 nm radiation (intensity ranged from 97.8–100.1 W/m^2^) for 3, 6, 9, 12, 18, 24, 27, 60 and 180 s, and 3, 5 and 10 min. At each evaluated time, a pool of five peel plugs was placed in a sterile filter bag (BagPage^®^, Interscience BagSystem, Saint Nom la Brétèche, France) by triplicate. The inoculated orange juice was placed in 12-well plates (Falcon, USA). Each well contained one millilitre of juice, with a depth of 0.4 cm. Orange juice was exposed to UV 254 nm radiation (intensity 100.9–107.9 W/m^2^) for 5, 10, 15, 20, 25, 30, 45 and 60 min, and three samples were taken at each time. The UV-C dose (kJ/m^2^) was calculated as the intensity measured (W/m^2^) × time (s)/1000 (Table 1). The assays were performed individually for each microorganism, and the experiments were performed twice on different working days.

### 2.5. Microbial Analysis

After the UV 254 nm treatments, each orange peel sample was diluted with 5 mL of buffered peptone water ((BPW), Biokar) and homogenised in a blender (IUL, Barcelona, Spain) for 90 s (250 impacts/min). The homogenates of orange peel plugs and the samples of orange juice were activated by heat shock (as described in Section 2.1), serially diluted in SP and spread on agar plates. *A. acidoterrestris* CECT 7094T and *A. acidoterrestris* TA-1.183 were plated on YSG agar and incubated at 45 °C for 24 h. *B. cereus* and *B. coagulans* samples were spread on NA plates and incubated at 30 °C for 24 h. 

### 2.6. Curve Fitting and Statistical Analysis

Inactivation data were expressed as log (N/N_0_) to avoid small differences in the initial concentrations between experiments. Survival curves were obtained by plotting the logarithm of the survival fraction (N/N_0_) versus treatment doses, expressed in kJ/m^2^. N is the spore count after UV-C exposition, and N_0_ is the initial number of spores before treatment. The GInaFiT software was used for modelling the fit of the survival curves [32]. As UV 254 nm inactivation curves obtained on fruit surface show tail, the Weibull model [33], the Weibull plus tail model [34] and the Biphasic model [35] were used and compared for each microorganism inactivation curve. On the contrary, UV 254 nm inactivation curves obtained in fruit juice do not show shoulder, neither do tail. Therefore, the Log-linear regression [36] and the Weibull model [33] were used and compared for each microorganism inactivation curve. 

The Log-linear regression, a model typically used to assess the effect of a wide array of processing factors on microbial inactivation, including UV 254 nm treatment, is described by Equation (2) [36]:

(2) $$log\;N=log\;N_{0}-\frac{k_{max}d}{\ln10}$$

where *k_max_* is the first-order inactivation rate constant (m^2^/kJ), and *d* is the applied UV 254 nm dose (kJ/m^2^). 

The Weibull model is described by Equation (3) [33]:

(3) $$log\;N=log\;N_{0}-{\left(\frac{d}{\delta }\right)}^{p}$$

where *d* is the applied UV dose (kJ/m^2^), *δ* (kJ/m^2^) is a scale parameter indicating the UV 254 nm dose for the first decimal reduction, and *p* (dimensionless) is a shape parameter describing concavity or convexity of the curve.

The Weibull plus tail model is described by Equation (4) [34]:

(4) $$log\;N=log\;\left[\left(N_{0}-N_{res}\right)\cdot 10^{\left(-{\left(\frac{d}{\delta }\right)}^{p}\right)}+N_{res}\right]$$

where *N_res_* represents the residual cell concentration where the tail starts, *d* is the applied UV dose (kJ/m^2^), *δ* (kJ/m^2^) is a scale parameter indicating the UV 254 nm dose for the first decimal reduction, and *p* (dimensionless) is a shape parameter.

The Biphasic model is described by Equation (5) [35]:

(5) $$log\;N=log\;N_{0}+log\;\left(f^{\left(-k_{max1}d\right)}+{\left(1-f\right)}^{\left(-k_{max2}d\right)}\right)$$

where *f* is the fraction of initially major subpopulation, *K_max_*_1_ and *K_max_*_2_ are the first-order inactivation rate constants for initially major and minor population (m^2^/kJ), and *d* is the applied UV 254 nm dose (kJ/m^2^).

Model performance and comparison were performed using the root mean square error (RMSE) and the adjusted correlation coefficient R^2^ (R^2^-adj). RMSE measures the average deviation between the observed and the fitted values. Therefore, the model with the smallest RMSE was considered to be the best fit for the inactivation curve. 

## 3. Results

### 3.1. Bacterial Spore Inactivation on Orange Peel

**Figure 1:**
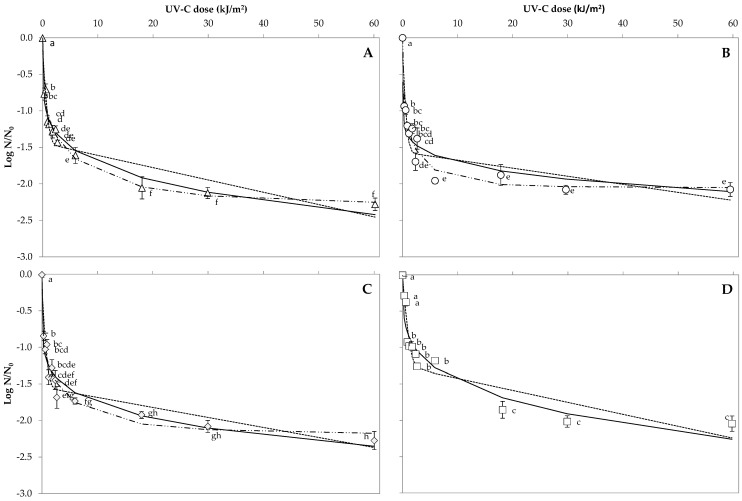
Survival curve of *A. acidoterrestris* CECT 7094T (**A**), *A. acidoterrestris* TA-1.183 (**B**), *B. cereus* (**C**) and *B. coagulans* (**D**) spores on orange peel at evaluated UV-C at 254 nm doses. The dots mean experimental data (black dots), and the lines represent fitted data predicted values, according to the Weibull model (solid line), the Biphasic model (dash-dot line) and the Weibull plus tail model (dashed line). Different letters indicate significant differences among spore reductions at different UV-C exposition doses, according to the student’s t-test (*p* < 0.05).

The initial population of *A. acidoterrestris* CECT 7094T, *A. acidoterrestris* TA-1.183, *B. cereus* and *B. coagulans* spores on orange peel was 5.07 ± 0.11, 4.92 ± 0.03, 4.80 ± 0.10 and 5.01 ± 0.09 log CFU/cm^2^, respectively. The reduction curves of the four bacterial spores artificially inoculated on orange peel and exposed to UV 254 nm light for 0 to 10 min were assessed (Figure 1). Table 1 shows the relation between the evaluated UV 254 nm exposition time and the UV 254 nm dose applied for each type of food matrix. After 10 min of UV 254 nm light exposition, corresponding to a UV 254 nm dose of 59.9 kJ/m^2^, *A. acidoterrestris* CECT 7094T, *A. acidoterrestris* TA-1.183, *B. cereus* and *B. coagulans* spores were reduced by 2.28 ± 0.21, 2.08 ± 0.23, 2.27 ± 0.30 and 2.05 ± 0.23 log, respectively. The number of *A. acidoterrestris* CECT 7094T spores on the orange peel showed a significant reduction after 0.3 kJ/m^2^ UV 254 nm dose (0.77 ± 0.2 log reduction); meanwhile, no significant differences in spore reduction were observed when the UV 254 nm dose increased from 18.0 to 59.9 kJ/m^2^, with spore reductions ranging from 2.06 ± 0.37 to 2.28 ± 0.21 log. *A. acidoterrestris* TA-1.183 spores also showed a significant reduction after exposure to a UV 254 nm dose of 0.3 kJ/m^2^ (0.94 ± 0.13 log reduction). When the treatment time was increased from 5.9 to 59.9 kJ/m^2^, *A. acidoterrestris* TA-1.183 spore counts on the orange peel were not significantly different with spore reductions of 1.96 ± 0.1, 1.88 ± 0.35, 2.05 ± 0.15 and 2.08 ± 0.23 log after 5.9, 18.0, 29.9 and 59.9 kJ/m^2^, respectively. *B. cereus* spores reached a significant reduction after 0.3 kJ/m^2^ (0.84 ± 0.16 log reduction), whereas 0.9 kJ/m^2^ of a UV 254 nm exposition were needed to obtain a significant reduction on *B. coagulans* spores on the peel (0.93 ± 0.13 log reduction). *B. cereus* spores on the peel reached 1.92 ± 0.12 log reduction after the exposition to 18.0 kJ/m^2^, a value that was not significantly different to the reductions observed after UV 254 nm doses of 29.9 and 59.9 kJ/m^2^ (2.08 ± 0.20 and 2.27 ± 0.30 log reductions, respectively). Similar results were obtained for *B. coagulans,* spore inactivation counts were not significantly different in a range of 18.0–59.9 kJ/m^2^, with reductions ranging from 1.86 ± 0.40–2.05 ± 0.37 log. 

### 3.2. Bacterial Spore Inactivation in Orange Juice

The orange juice used in the spore inactivation trials had a pH value of 3.31 ± 0.01. TA was 11.2 ± 0.1 g citric acid/L, and SSC was 11.77 ± 0.06 °Brix. The juice UV light transmittance measured at 254 nm was 0.15%.

**Figure 2:**
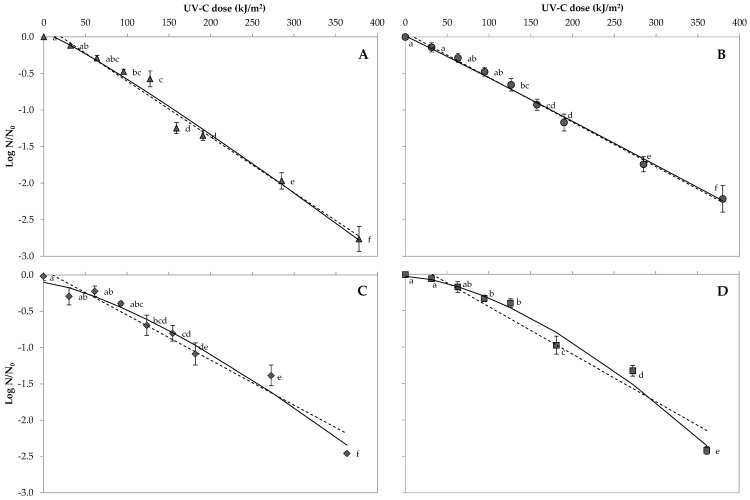
Survival curve of *A. acidoterrestris* CECT 7094T (**A**), *A. acidoterrestris* TA-1.183 (**B**), *B. cereus* (**C**) and *B. coagulans* (**D**) spores in orange juice at evaluated UV-C at 254 nm doses. The dots mean experimental data (black dots), and the lines represent fitted data predicted values, according to the Log-linear model (dashed line) and the Weibull model (solid line). Different letters indicate significant differences among spore reductions at different UV-C exposition doses according to the student’s t-test (*p* < 0.05).

Figure 2 shows the reduction curves of the four bacterial spores exposed to UV 254 nm in orange juice. The artificially inoculated orange juice was exposed to UV 254 nm light for different times, from 0 to 60 min (Table 1). The initial population of *A. acidoterrestris* CECT 7094T, *A. acidoterrestris* TA-1.183, *B. cereus* and *B. coagulans* spores was 5.23 ± 0.25, 5.13 ± 0.03, 4.21 ± 0.30 and 4.76 ± 0.46 log CFU/mL, respectively. After the higher exposition to UV 254 nm light (370.6 kJ/m^2^), reductions were 2.76 ± 0.42, 2.22 ± 0.45, 2.46 ± 0.05 and 2.42 ± 0.42 log for *A. acidoterrestris* CECT 7094T, *A. acidoterrestris* TA-1.183, *B. cereus* and *B. coagulans* spores, respectively. *A. acidoterrestris* CECT 7094T spores in the orange juice showed a significant reduction after a UV 254 nm exposition of 94.4 kJ/m^2^ (0.48 ± 0.09 log reduction). The number of spores in the juice decreased drastically after 157.2 kJ/m^2^ UV 254 nm treatment (1.25 ± 0.18 log reduction). A significant reduction of *A. acidoterrestris* TA-1.183 spores in the juice was observed after UV 254 nm treatment of 125.9 kJ/m^2^ (0.66 ± 0.21 log reduction). The spore counts decreased with a higher exposition time, with reductions of 1.17 ± 0.28 and 1.74 ± 0.25 log after UV 254 nm doses of 185.8 and 278.8 kJ/m^2^, respectively. After a UV 254 nm exposition of 125.9 kJ/m^2^, *B. cereus* spore population was significantly reduced by 0.69 ± 0.34 log units, whereas *B. coagulans* spore numbers were reduced by 0.33 ± 0.11 log after 94.4 kJ/m^2^. In all of the evaluated bacterial spores, the increased dose of UV 254 nm decreased the spore population in juice until the maximum evaluated time (60 min) equivalent to 370.6 kJ/m^2^. No changes in juice taste or colour were observed after the longest time tested (60 min).

### 3.3. Predictive Microbial Models and Their Comparison on Orange Peel

In order to analyse the goodness of fit, Table 2 shows the RMSE and the R_2_-adj of the Weibull model, the Weibull plus tail model and the Biphasic model estimating bacterial spore reduction on orange peel after UV-C at 254 nm treatment. The UV-resistance parameters obtained from fitting the evaluated inactivation models are shown in Table 3. 

The Weibull model accurately described all the inactivation curves, with R^2^-adj values of 0.717–0.887 (Table 2). All the inactivation curves showed *p* values lower than 1 (Table 3), which was in accordance with the notorious upward concavity observed. *A. acidoterrestris* TA-1.183 needed the lower dose for the first decimal reduction (*δ* = 0.17 ± 0.12), followed by *B. cereus* (*δ* = 0.27 ± 0.18), *A. acidoterrestris* CECT 7094T (*δ* = 0.54 ± 0.26) and finally *B. coagulans,* which needed the highest dose (*δ* = 1.58 ± 0.77).

Regarding the Weibull plus tail model, R^2^-adj values of 0.838–0.900 were observed, which means that it adequately characterized the bacterial spore inactivation curves. Similar to the Weibull model, all the curves showed *p* values < 1 according to the upward concavity. In comparison to the Weibull model, higher doses of radiation were needed to achieve the first decimal reduction. *δ* values were 0.44 ± 0.16, 0.45 ± 0.19, 0.75 ± 0.25 and 1.58 ± 0.78 for *A. acidoterrestris* TA-1.183, *B. cereus*, *A. acidoterrestris* CECT 7094T and *B. coagulans*, respectively. 

The Biphasic model was appropriate for representing survival data of *A. acidoterrestris* CECT 7094T, *A. acidoterrestris* TA-1.183, *B. cereus* and *B. coagulans* with R^2^-adj values of 0.845, 0.782, 0.821 and 0.849, respectively. From the total spore population on the orange peel, a high proportion of spores sensitive to UV 254 nm were observed for the four bacteria studied, as *f* values ranged between 0.94 and 0.95. The sensitive population of *A. acidoterrestris* TA-1.183 showed the highest speed of decrease (*k_max_*_1_ = 3.44 ± 0.55 m^2^/kJ); meanwhile, the sensitive population of *B. coagulans* showed the lowest speed of decrease (*k_max_*_1_ = 2.09 ± 0.33 m^2^/kJ). The inactivation rate of the resistant population was similar for all the bacteria tested.

The goodness of fit of the three evaluated models for the four bacterial spore inactivation curves on orange peel can be evaluated throughout the comparison of RMSE values. The Weibull plus tail model showed the best performance when modelling inactivation curves of spore inactivation of both *A. acidoterrestris* strains and *B. cereus*, as it was the model that showed the lowest RMSE. However, the Biphasic model showed the best performance characterizing *B. coagulans* spore inactivation curves. 

### 3.4. Predictive Microbial Models and Their Comparison in Orange Juice

The statistical indices of the Log-linear model and the Weibull model estimating bacterial spore reduction in orange juice after UV-C exposure are shown in Table 4, and the UV-resistance parameters obtained from the fitting of different evaluated inactivation models are shown in Table 5.

The Log-linear model accurately described all the inactivation curves of the studied spores in orange juice, with R_2_-adj values of 0.850–0.932. The inactivation rate of bacterial spores in orange juice ranged from 0.01–0.02 m^2^/kJ for the four bacteria tested.

The Weibull model was appropriate for representing the survival data of *A. acidoterrestris* CECT 7094T, *A. acidoterrestris* TA-1.183, *B. cereus* and *B. coagulans*, as it exhibited a high R^2^-adj value 0.933, 0.904, 0.863 and 0.933, respectively. The observed *p* values were higher than 1 for all spore inactivation curves, showing a downward concavity. *A. acidoterrestris* CECT 7094T needed the lower dose for the first decimal reduction (*δ* = 147.5 ± 14.59), followed by *A. acidoterrestris* CECT 7094T (*δ* = 172.05 ± 19.07), *B. cereus* (*δ* = 201.38 ± 17.08) and finally *B. coagulans,* that needed the highest dose (*δ* = 212.98 ± 12.45).

In order to analyse the goodness of fit of the two predictive microbial models evaluated, the corresponding values of RMSE were compared. The Log-linear model showed the lowest RMSE value for *A. acidoterrestris* TA-1.183, whilst the Weibull model showed the lowest RMSE values for *A. acidoterrestris* CECT 7094T, *B. cereus* and *B. coagulans.*

## 4. Discussion

Spore-forming bacteria are one of the main concerns for fruit juice processors, as spores can survive thermal and HPP treatments that are usually applied in the food industry. The main source of bacterial spores in the fruit juice industry is the field soil where fruits are cultivated. Therefore, cleaning and disinfecting fruits before juice processing could help to reduce bacterial spores in the juice. Once bacterial spores reach the juice, alternative methods to thermal and HPP treatments are needed. UV-C radiation has been shown previously to reduce bacterial spores in some fruit juices. In this study, the efficacy of UV-C radiation at 254 nm to eliminate the spores of *A. acidoterrestris*, *B. coagulans* and *B. cereus* on orange peel and in orange juice has been evaluated to simulate UV-C treatment of whole oranges at their arrival to the food industry and UV-C treatment of the final product (juice).

According to the data obtained, a three-minute treatment (18.0 kJ/m^2^) could be enough to significantly reduce the spore numbers on the orange peel (around 2 log units). Incrementing treatment time did not significantly enhance UV 254 nm treatment efficacy. Little information about microorganism’s inactivation on a fruit surface by UV-C light is found in the literature. Gündüz et al. [37] evaluated the germicidal effect of UV-C light (0.26–15.84 kJ/m^2^) on citrus fruit inoculated with *Penicillium* spp. spores by spot method. Maximum reductions of 2.75 log CFU/orange of *Penicillium digitatum* were obtained at the UV-C dose of 3.17 kJ/m^2^. However, previous studies suggested that fungi are more resistant to UV-C light than bacterial spores due to their size [23].

For all bacterial spores studied, 15–20 min UV 254 nm treatment (94.4–125.9 kJ/m^2^) were needed to significantly reduce the initial spore counts in orange juice, and more than 45 min (278.8 kJ/m^2^) was needed to achieve a spore reduction higher than 2 log units. Tremarin et al. [21] observed a higher *A. acidoterrestris* spores reduction in apple juice, 5 log after 8 min of exposure to UV-C (13.44 W/m^2^). This difference could be attributed to the higher transmittance of apple juice (27%) compared to the transmittance of the orange juice used in this experiment (0.15%). On ‘Cantaloupe’ melon juice, 20 min of exposure at the same UV-C radiation intensity (13.44 W/m^2^) were required for a 4.7 log unit reduction of the *A. acidoterrestris* spores [26]. The authors explained it by the differences in transmittance of the juices, which was significantly lower in the case of melon (around 10%). This hypothesis is supported by the studies carried out by Baysal et al. [25]: a 5.5 log reduction of the *A. acidoterrestris* spores in grape juice was achieved after 15 min of UV-C treatment (1.31 W/m^2^), whereas spores in apple juice were only reduced by 2.1 log reduction under the same treatment conditions. In this case, the difference in efficacy of UV between the juices was also attributed to the lower absorption coefficient of grape juice when compared to apple juice. Higher reductions of the *A. acidoterrestris* spores in apple juice were observed by Sauceda-Gálvez et al. [38] by 5.8 log CFU/mL after a dose of 21.5 J/mL. Prado et al. [27] reported that exposure of *A. acidoterrestris* spores to UV-C (4.2–12.6 kJ/m^2^) in orange juice (2% transmittance) reduced their population until 3 log units. Lower *A. acidoterrestris* spores reduction after orange juice (2% transmittance) treatment with UV-C light were reported by Ferreira et al. [39], only 1 log unit after a 12.6 kJ/m^2^ treatment. 

Although there are several studies about UV-C light efficacy in reducing bacterial spores in fruit juices, their comparison is quite risky due to the different transmittance of the food matrix, the depth of the juice treated and the UV-C equipment. For example, Baysal et al. [25] tested spore inactivation in juice at 0.15 cm depth, while in the present study, a higher depth was evaluated (0.4 cm).

Spore inactivation curves after the UV 254 nm treatment of all tested species on orange peel showed tails, which suggests varying levels of resistance, possibly caused by mixed populations or aggregation of microorganisms. However, according to the *f* values obtained from the Biphasic model, 94–95% of the total spores were sensitive to UV 254 nm light. The curves showed an upward concavity, which is reflected in *p* values obtained in the Weibull and the Weibull plus tail models. This indicates that the treatment efficacy was reduced at higher doses. From the results obtained, it can be concluded that *A. acidoterrestris* TA-1.183 spores were the most sensitive to UV 254 nm treatment of orange peels, as they showed the lowest *δ* values from the Weibull and the Weibull plus tails models and the highest speed of decrease of the sensitive population (*k_max_*_1_), according to the Biphasic model. The *A. acidoterrestris* CECT 7094T and *B. cereus* spores showed intermediate resistance to UV 254 nm; meanwhile, the *B. coagulans* spores were the most resistant with the highest *δ* values from the Weibull and the Weibull plus tails models and the lowest speed of decrease of the sensitive population (*k_max_*_1_), according to the Biphasic model. 

In orange juice, the spore inactivation curves did not show shoulders or tails. The obtained curves were almost linear for the *A. acidoterrestris* TA-1.183 spores or with downward concavity for the rest of bacterial spores tested, according to the *p* values of the Weibull model. Similar to the results observed with orange peel, the *B. coagulans* spores in orange juice were the most resistant to UV-C and showed the highest *δ* values, according to the Weibull model. However, in orange juice, the most sensitive populations were the *A. acidoterrestris* CECT 7094T spores, with the lowest *δ* values and the highest speed of decrease according to the Log-linear model. 

As it has been observed in the present study and as it has been previously described, the inactivation of microorganisms with UV-C treatment can show different shapes, from first-order kinetics [40] to sigmoidal shapes with shoulders and/or tails [41]. For instance, the *A. acidoterrestris* spore inactivation by UV-C light in white grape juice fitted to the Log-linear plus tail and Weibull models, while the inactivation data in apple juice only fitted Log-linear plus tail model [25]. However, the Log-linear plus tail model showed the best goodness of fit in terms of RMSE, R^2^ and R^2^-adj. The *A. acidoterrestris* spore inactivation curves in apple juice obtained by Tremarin et al. [21,42] followed a first-order kinetic model. *B. cereus* and *B. coagulans* spore survival curves after UV-C treatment in a buffer with an absorption coefficient of 11.1 cm^−1^ fitted a Log-linear plus shoulder model [28]. The authors did not report differences among *K_max_* values among the species, but the shoulder length widely changed, and therefore, the UV-C resistance was due to changes in shoulder length. Similar to the results obtained in the present study, Gayán et al. [28] reported a higher resistance of *B. coagulans* when compared to *B. cereus*. In the case of non-sporulated bacteria, Fenoglio et al. [43] observed that *Escherichia coli*, *Lactobacillus plantarum* and *Saccharomyces cerevisae* UV-C inactivation curves could be appropriately represented by the Weibull, Coroller and Biphasic models. 

According to Setlow [44], bacterial spores are 20- to 50-fold more resistant to UV radiation than growing cells, depending on the bacterial species and UV wavelength. Spore resistance to UV is caused by a change in the DNA’s UV photochemistry due to the binding of α/β-type acid-soluble spore proteins (SASPs) and DNA repair during spore outgrowth, with this repair catalysed in part by spore enzymes [44,45]. In addition, some pigments in the outer layers of spores are important in protecting them from UV damage, and spores’ huge dipicolinic acid (DPA) depot also influences their UV resistance [46]. Regarding UV light mode of action, the inactivation of cells by UV light is mainly due to the fatal effect on DNA. In addition, it causes other effects such as abnormal ion flow, increased cell membrane permeability and depolarization of cell membrane [47]. Prado et al. [27] observed that *A. acidoterrestris* spores subjected to 12.6 kJ/m^2^ UV-C light treatment showed visible changes, such as morphological distortions, central depression and expressive roughness, when observed with a scanning electron microscopy.

## 5. Conclusions

As it has been demonstrated, UV-C light at 254 nm is capable of reducing the bacterial spores tested in both orange peel and orange juice. However, in fruit juices, UV-C efficacy depends on the juice characteristics (mainly light transmittance) because of the low-penetration capacity of UV-C light. Therefore, establishing a standard treatment for juices may be difficult, as light penetration is matrix-dependent (i.e., fruit, juice type of processing/clarification, presence of pulp). 

To the best of our knowledge, this is the first report about bacterial spore inactivation on a fruit peel using UV-C light, and we have demonstrated that it is more effective in reducing bacterial spores on orange peels than in orange juice. As raw fruits are the main source of bacterial spores in fruit juices, reducing bacterial spores on fruit peels could, consequently, reduce their entrance into the processing plants. Therefore, implementing a UV-C treatment for the whole fruit before processing could improve the safety and quality of fruit juices.

## Figures and Tables

**Table 1:** The relation between the evaluated UV 254 nm exposition times and the UV 254 nm doses applied (kJ/m^2^) for each type of food matrix (orange or juice).

Orange Peel Exposition		Orange Juice Exposition	
Time (seconds)	UV 254 nm (kJ/m^2^)	Time (min)	UV 254 nm (kJ/m^2^)
3	0.3 ± 0.0	5	31.4 ± 0.4
6	0.6 ± 0.0	10	62.8 ± 0.8
9	0.9 ± 0.0	15	94.4 ± 1.2
12	1.2 ± 0.0	20	125.9 ± 1.5
18	1.8 ± 0.0	25	157.2 ±2.0
24	2.4 ± 0.0	30	185.8 ± 2.3
27	2.7 ± 0.0	45	278.8 ± 2.8
60	5.9 ± 0.0	60	370.6 ± 5.5
180	18.0 ± 0.1		
300	29.9 ± 0.4		
600	59.9 ± 0.2		

Each value is the mean ± standard deviation.

**Table 2:** The statistical indices of the Weibull model, the Weibull plus tail model and the Biphasic model estimating the bacterial spore reduction on orange peel during UV-C at 254 nm treatment.

Bacterial Spore	Statistical Indices	Weibull Model	Weibull Plus Tail Model	Biphasic Model
*A. acidoterrestris* CECT 7094T	RMSE	0.220	0.207	0.257
	R^2^-adj	0.887	0.900	0.845
*A. acidoterrestris* TA-1.183	RMSE	0.324	0.223	0.284
	R^2^-adj	0.717	0.866	0.782
*B. cereus*	RMSE	0.250	0.241	0.2702
	R^2^-adj	0.847	0.858	0.821
*B. coagulans*	RMSE	0.264	0.266	0.257
	R^2^-adj	0.840	0.838	0.849

RMSE: root mean squared error and R^2^-adj: coefficient of determination adjusted.

**Table 3:** UV-resistance parameters obtained from the fitting of Weibull model, Weibull plus tail model and Biphasic model to inactivation curves of different bacterial spores in orange peel.

Model	Kinetic Parameters	*A. acidoterrestris* CECT 7094T	*A. acidoterrestris* TA-1.183	*B. cereus*	*B. coagulans*
Weibull	*δ*	0.54 (0.26)	0.17 (0.12)	0.27 (0.18)	1.58 (0.77)
	*p*	0.19 (0.02)	0.14 (0.01)	0.16 (0.02)	0.23 (0.02)
Weibull plus tail	*δ*	0.75 (0.25)	0.44 (0.16)	0.45 (0.19)	1.58 (0.78)
	*p*	0.28 (0.03)	0.30 (0.05)	0.26 (0.04)	0.23 (0.04)
Biphasic	*f*	0.94 (0.01)	0.95 (0.01)	0.95 (0.01)	0.94 (0.01)
	*k_max_*_1_	2.80 (0.43)	3.44 (0.55)	2.94 (0.44)	2.09 (0.33)
	*k_max_*_2_	0.04 (0.00)	0.03 (0.01)	0.03 (0.00)	0.04 (0.01)

Values in brackets represent the standard error of each parameter. *δ*: dose for the first decimal reduction (kJ/m^2^), *p*: shape parameter (dimensionless), *f*: fraction of initially major subpopulation (major subpopulation is the least resistant of both) and *K_max_*_1_ and *k_max_*_2_ (m^2^/kJ): inactivation rates.

**Table 4:** The statistical indices of the Log-linear model and the Weibull model estimating bacterial spore reduction in orange juice during UV-C at 254 nm treatment.

Bacterial Spore	Statistical Indices	Log-Linear Model	Weibull Model
*A. acidoterrestris* CECT 7094T	RMSE	0.236	0.234
	R^2^-adj	0.932	0.933
*A. acidoterrestris* TA-1.183	RMSE	0.226	0.229
	R^2^-adj	0.907	0.904
*B. cereus*	RMSE	0.284	0.271
	R^2^-adj	0.850	0.863
*B. coagulans*	RMSE	0.252	0.209
	R^2^-adj	0.902	0.933

RMSE: root mean squared error and R^2^-adj: coefficient of determination adjusted.

**Table 5:** UV-resistance parameters obtained from the fitting of the Log-linear model and the Weibull model to inactivation curves of different bacterial spores in orange juice.

Model	Kinetic Parameters	*A. acidoterrestris* CECT 7094T	*A. acidoterrestris* TA-1.183	*B. cereus*	*B. coagulans*
Log-linear	*k_max_*	0.02 (0.00)	0.01 (0.00)	0.01 (0.00)	0.01 (0.00)
Weibull	*δ*	147.5 (14.59)	172.05 (19.07)	201.38 (17.08)	212.98 (12.45)
	*p*	1.11 (0.10)	1.03 (0.11)	1.37 (0.17)	1.60 (0.15)

Values in brackets represent the standard error of each parameter. *K_max_*: inactivation rate (m^2^/kJ), *δ*: dose for the first decimal reduction (kJ/m^2^) and *p*: shape parameter (dimensionless).

## Data Availability

The data presented in this study are available on request from the corresponding author.
